# Petroleum Benzine as an Anæsthetic

**Published:** 1896-12

**Authors:** 


PETROLEUM BENZINE AS AN ANÆSTHETIC. In a report issued by the kingdom of Saxony in relation to veterinary medicine, a notice by Müller occurs regarding the use of petroleum benzine as an anæsthetic. Shortly after inhalation a three-year-old dog showed great liveliness, frequent respiration, irregular pulse, mydriasis, cyanosis, and trembling in the muscles. Finally, a general cramp or convulsion, and after twenty minutes a certain degree of narcosis interrupted with cramps. On suspending the inhalation, when about 70 grains had been administered, consciousness speedily returned The temperature varied during the experiment from 38° to 40°. A dog which was suffering from heart-trouble fell into a state of coma at the beginning of inhalation. Total loss of consciousness followed the administration of 600 grains in the case of an old horse, after which an attack of general cramp followed; the circulation and respiration were not markedly influenced.—*Veeartsenijk. Bladen*, Deel ix., Heft 4.

